# Supplementary material for: Adrenergic activation modulates the signal from the Reissner fiber to cerebrospinal fluid-contacting neurons during development
Source: eLife. 2020 Oct 13;9:e59469. doi: 10.7554/eLife.59469 (PMC7591253; doi:10.7554/eLife.59469)
Supplement: Transparent reporting form [file elife-59469-transrepform.docx]

***eLife’s* transparent reporting form**

We encourage authors to provide detailed information *within their submission* to facilitate the interpretation and replication of experiments. Authors can upload supporting documentation to indicate the use of appropriate reporting guidelines for health-related research (see [EQUATOR Network](http://www.equator-network.org/%20)), life science research (see the [BioSharing Information Resource](https://biosharing.org/)), or the [ARRIVE guidelines](http://www.plosbiology.org/article/info:doi/10.1371/journal.pbio.1000412) for reporting work involving animal research. Where applicable, authors should refer to any relevant reporting standards documents in this form.

If you have any questions, please consult our Journal Policies and/or contact us: [editorial@elifesciences.org](mailto:editorial@elifesciences.org).

**Sample-size estimation**

- You should state whether an appropriate sample size was computed when the study was being designed
- You should state the statistical method of sample size computation and any required assumptions
- If no explicit power analysis was used, you should describe how you decided what sample (replicate) size (number) to use

Please outline where this information can be found within the submission (e.g., sections or figure legends), or explain why this information doesn’t apply to your submission:

In the material and method section, we state :

" For all but one experiment (Figure 4D), we used at least 3 biological replicates, sometimes more (see individual figure legends), to reach an acceptable level of reproducibility of results while minimizing the number of animals used, in accordance with the "reduce" 3R rule."

Please note that in the case of Figure 4D, we were limited in the number of biological replicates by the COVID-19 lockdown. This will be solved in the coming weeks.

**Replicates**

- You should report how often each experiment was performed
- You should include a definition of biological versus technical replication
- The data obtained should be provided and sufficient information should be provided to indicate the number of independent biological and/or technical replicates
- If you encountered any outliers, you should describe how these were handled
- Criteria for exclusion/inclusion of data should be clearly stated
- High-throughput sequence data should be uploaded before submission, with a private link for reviewers provided (these are available from both GEO and ArrayExpress)

Please outline where this information can be found within the submission (e.g., sections or figure legends), or explain why this information doesn’t apply to your submission:

The number of biological replicates is fully described in each figure legend (and in Figure 3–figure supplement 1, as stated in the figure legend).

Technical replicates were only carried out for qRT-PCR, as stated in the material and methods section. The average of the technical replicates was used for further analysis, precluding any artificial inflation of the replicates number.

In the material and method section, we state :

"No outlier value was excluded from our analysis"

**Statistical reporting**

- Statistical analysis methods should be described and justified
- Raw data should be presented in figures whenever informative to do so (typically when N per group is less than 10)
- For each experiment, you should identify the statistical tests used, exact values of N, definitions of center, methods of multiple test correction, and dispersion and precision measures (e.g., mean, median, SD, SEM, confidence intervals; and, for the major substantive results, a measure of effect size (e.g., Pearson's r, Cohen's d)
- Report exact p-values wherever possible alongside the summary statistics and 95% confidence intervals. These should be reported for all key questions and not only when the p-value is less than 0.05.

Please outline where this information can be found within the submission (e.g., sections or figure legends), or explain why this information doesn’t apply to your submission:

The statistical tests used, exact values of N, definitions of center and dispersion are specified in each figure legends. For the RNAseq and qRT-PCR, the precise data analysis and statistical approach, including multiple test correction, are detailed in the material and method section.

All figures show raw data as single points, in addition to the mean or median, as stated in the figure legends.

(For large datasets, or papers with a very large number of statistical tests, you may upload a single table file with tests, Ns, etc., with reference to sections in the manuscript.)

**Group allocation**

- Indicate how samples were allocated into experimental groups (in the case of clinical studies, please specify allocation to treatment method); if randomization was used, please also state if restricted randomization was applied
- Indicate if masking was used during group allocation, data collection and/or data analysis

Please outline where this information can be found within the submission (e.g., sections or figure legends), or explain why this information doesn’t apply to your submission:

- The design of experimental groups (designed according to the genotype and/or pharmacological treatment) is specified in the results section and detailed in the material and method section
- Masking was used for data analysis in Figure 2C, Figure 3D, Figure 4D-4E, Figure 2-figure supplement 1B-D

**Additional data files (“source data”)**

- We encourage you to upload relevant additional data files, such as numerical data that are represented as a graph in a figure, or as a summary table
- Where provided, these should be in the most useful format, and they can be uploaded as “Source data” files linked to a main figure or table
- Include model definition files including the full list of parameters used
- Include code used for data analysis (e.g., R, MatLab)
- Avoid stating that data files are “available upon request”

Please indicate the figures or tables for which source data files have been provided:

- Additional numerical source date files are provided for each figure.
- MATLAB codes used for data analysis are available at Github <https://github.com/wyartlab/Cantaut-Belarif-et-al.-2020>
- The list of the most significantly modified genes from the RNAseq experiment is provided as a supplementary table. The raw RNA-seq data have been deposited in the ArrayExpress database at EMBL-EBI ([www.ebi.ac.uk/arrayexpress](http://www.ebi.ac.uk/arrayexpress)) under accession number **E-MTAB-9615**.
